# The *in vitro* Photoinactivation of *Helicobacter pylori* by a Novel LED-Based Device

**DOI:** 10.3389/fmicb.2020.00283

**Published:** 2020-02-21

**Authors:** Paola Morici, Antonella Battisti, Giuseppe Tortora, Arianna Menciassi, Giovanni Checcucci, Francesco Ghetti, Antonella Sgarbossa

**Affiliations:** ^1^Nanoscience Institute, CNR and NEST, Scuola Normale Superiore, Pisa, Italy; ^2^The BioRobotics Institute, Polo Sant’Anna Valdera, Scuola Superiore Sant’Anna, Pontedera, Italy

**Keywords:** *Helicobacter pylori*, antimicrobial PDT, LEDs, porphyrins, flavins

## Abstract

The rise of antibiotic resistance is the main cause for the failure of conventional antibiotic therapy of *Helicobacter pylori* infection, which is often associated with severe gastric diseases, including gastric cancer. In the last years, alternative non-pharmacological approaches have been considered in the treatment of *H. pylori* infection. Among these, antimicrobial PhotoDynamic Therapy (aPDT), a light-based treatment able to photoinactivate a wide range of bacteria, viruses, fungal and protozoan parasites, could represent a promising therapeutic strategy. In the case of *H. pylori*, aPDT can exploit photoactive endogenous porphyrins, such as protoporphyrin IX and coproporphyrin I and III, to induce photokilling, without any other exogenous photosensitizers. With the aim of developing an ingestible LED-based robotic pill for minimally invasive intragastric treatment of *H. pylori* infection, it is crucial to determine the best illumination parameters to activate the endogenous photosensitizers. In this study the photokilling effect on *H. pylori* has been evaluated by using a novel LED-based device, designed for testing the appropriate LEDs for the pill and suitable to perform *in vitro* irradiation experiments. Exposure to visible light induced bacterial photokilling most effectively at 405 nm and 460 nm. Sub-lethal light dose at 405 nm caused morphological changes on bacterial surface indicating the cell wall as one of the main targets of photodamage. For the first time endogenous photosensitizing molecules other than porphyrins, such as flavins, have been suggested to be involved in the 460 nm *H. pylori* photoinactivation.

## Introduction

*Helicobacter pylori* is the major causative agent of gastric chronic infection leading to severe gastric diseases such as atrophic gastritis, peptic ulcer, MALT (Mucosa-Associated Lymphoid Tissue) lymphoma, and significantly increasing the risk of developing gastric adenocarcinoma. *H. pylori* infection represents a risk factor in almost 90% of cases of gastric cancer, which is the third leading cause of cancer-related deaths worldwide according to World Health Organization (Wroblewski et al., 2010; Moss, 2016; Burkitt et al., 2017). In fact, the International Agency for Research on Cancer has classified *H. pylori* as a group I carcinogen (World Health Organization [WHO] and International Agency for Research on Cancer [IARC], 2019). The clinical treatment involves the use of several antimicrobials such as clarithromycin, amoxicillin and levofloxacin or metronidazole with a combined intake of a proton pump inhibitor. However, the alarming antibiotic resistance in *H. pylori* is the main reason for the failure of this conventional therapy. As a consequence, many research efforts have been devoted to find alternative non-antibiotic therapies (Bush et al., 2011).

PhotoDynamic Therapy (PDT), largely employed as a clinical treatment for several malignant and premalignant pathologies, has gained importance also as a promising antimicrobial approach (Dougherty, 2002; Kharkwal et al., 2011; Van Straten et al., 2017; Cieplik et al., 2018; Ferreira Dos Santos et al., 2019; Quilbe et al., 2020). Antimicrobial PhotoDynamic Therapy (aPDT) relies on the application of a photosensitizer able to absorb appropriate wavelengths in the visible light range and to react with oxygen molecules inside and around cells, resulting in the production of singlet oxygen or other cytotoxic reactive oxygen species (ROS), which lead to cell death, after inducing photodamage. aPDT can efficiently kill a wide range of bacteria (both antibiotic-susceptible and multi-resistant strains), viruses, fungal, and protozoan parasites (Smijs and Pavel, 2011; Thomas et al., 2015; Wang et al., 2017; Alves et al., 2018; Andrade et al., 2018; Namvar et al., 2019) without causing development of resistance (Al-Mutairi et al., 2018; Ma et al., 2018). This approach is particularly advantageous against bacteria naturally producing and accumulating endogenous photosensitizers such as porphyrins and flavins (Plavskii et al., 2018), physiologically involved in several essential biological functions (e.g., respiration, biological oxidation, photosynthesis, sulfate reduction, metabolism of fats, carbohydrates, and proteins) (Shu et al., 2013; García-Angulo, 2017; Sepúlveda Cisternas et al., 2018). Several studies determined the presence of endogenous photosensitizing porphyrins and/or flavins in *Pseudomonas aeruginosa* (Dai et al., 2013; Wang et al., 2016), *Acinetobacter baumannii* (Zhang et al., 2014; Wang et al., 2016), *Candida albicans* (Zhang et al., 2016), *Aggregatibacter actinomycetemcomitans* (Cieplik et al., 2014; Fyrestam et al., 2015), methicillin-resistant *Staphylococcus aureus* (Biener et al., 2017), *Porphyromonas gingivalis* (Fyrestam et al., 2015; Yoshida et al., 2017), *Saccharomyces cerevisiae* (Fyrestam et al., 2015; Hoenes et al., 2018), *Legionella rubrilucens* (Schmid et al., 2019), and *Neisseria gonorrhoeae* (Wang et al., 2019). The susceptibility to PDT has been demonstrated in *Propionibacterium acnes*, the etiological agent of acne, which produces porphyrins exploitable as photosensitizers (Romiti et al., 2000; Ashkenazi et al., 2003).

It has long been known that *H. pylori* also spontaneously produces porphyrins, making it a suitable target for PDT (Hamblin et al., 2005). In previous studies, we have analyzed the composition of *H. pylori* endogenous porphyrins and their photophysical characteristics in bacterial extracts as well as within planktonic and biofilm growing cells. *H. pylori* accumulates a porphyrin mixture mainly composed by protoporphyrin IX (PPIX) and coproporphyrin I and III (CPI and CPIII). The absorption spectrum of the mixture shows a main peak at 405 nm with the characteristic porphyrin absorption bands at longer wavelengths (Battisti et al., 2017a, b). Preliminary clinical studies have been conducted on *H. pylori* infected patients by administering a porphyrin precursor and blue light through laser endoscopy, highlighting a significant reduction of *H. pylori* in biopsies after the treatment (Wilder-Smith et al., 2002). Later, other authors have shown remarkable reduction of antrum bacterial load after endoscopic treatment with a violet light source only (Ganz et al., 2005; Lembo et al., 2009). The possible collateral effects of aPDT on healthy gastric mucosa have also been investigated: the phototreatments did not cause side effects, even when the release of porphyrins from bacteria in the surrounding tissues was simulated (Faraoni et al., 2018). Nevertheless, the endoscopic treatment is invasive and associated to a poor patient compliance. Thus, alternative devices have been taken into account (Li et al., 2016; Romano et al., 2016). In the CapsuLight project, a swallowable pill containing LED sources has been proposed to perform intragastric *H. pylori* phototherapy in a minimally invasive way (Tortora et al., 2016). For this purpose it is crucial to determine the best illumination parameters able to activate the *H. pylori* photosensitizers, and to evaluate the LED emission module before proceeding to the integration in the final ingestible device, in order to maximize the bacteria photokilling during aPDT while taking care of battery power needs. Hence, the present study is aimed at evaluating the bactericidal effect of aPDT on *H. pylori* strains by using a novel LED-based device, developed to perform *in vitro* irradiation tests on bacterial cultures, as a first necessary step for further *in vivo* studies.

## Materials and Methods

### Bacterial Strains and Cultivation Conditions

Two bacterial strains, purchased from LGC Standards S.r.l. (Milan, Italy), were used in this study: a laboratory-adapted strain, ATCC 43504, and a virulent strain (*cag*A+ and *vac*A+), ATCC 700824 (J99). Both strains were stored at −80°C in Brucella Broth (BB, Thermo Fisher Scientific Remel Products, Lenexa, KS, United States) supplemented with 10% (v/v) heat-inactivated fetal bovine serum (FBS, Gibco, Life Technologies, Carlsbad, CA, United States) and 20% (v/v) glycerol. From frozen stocks, bacteria were cultured overnight in BB supplemented with 10% (v/v) FBS at 37°C in a microaerophilic atmosphere (CampyGen Compact, Oxoid, Hampshire, United Kingdom) with shaking at 170 rpm in the dark.

After incubation, *H. pylori* cells were harvested in mid-log growth phase by centrifugation at 4000 × *g* for 10 min, washed to remove trace amounts of culture medium, and to avoid the influence of medium pigments. Bacterial suspensions at a concentration of 2 × 10^6^ CFU/ml in Phosphate-Buffered Saline (PBS; pH 7.4) were prepared for photoinactivation assays.

### LED-Based Device and Lighting Conditions for the Photoinactivation Assays

**FIGURE 1:**
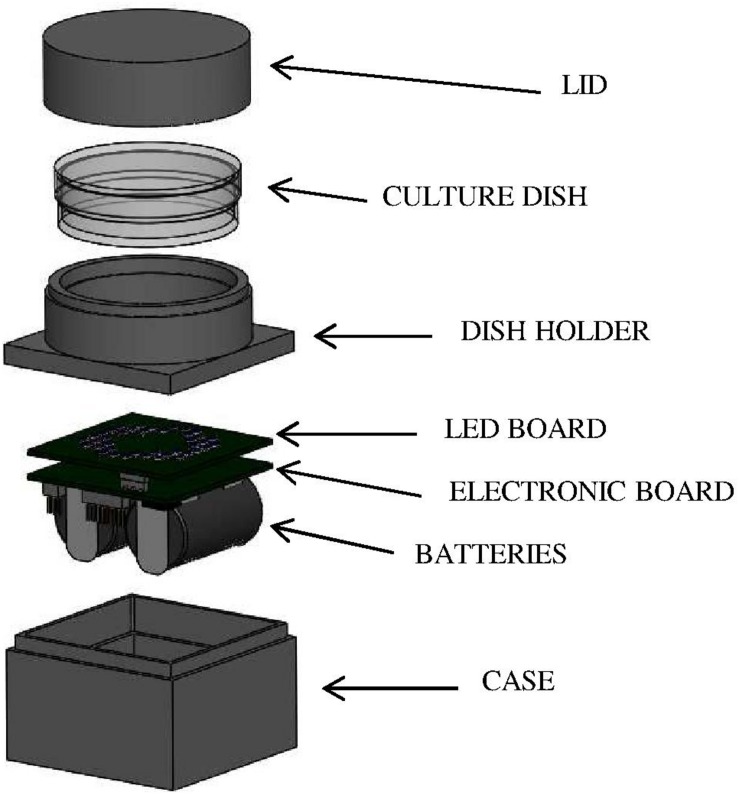
LED-based device for *in vitro* irradiation tests. LEDs were mounted on a board (LED BOARD) coupled with a programmable microcontroller (ELECTRONIC BOARD); two lithium BATTERIES provide suitable power according to experimental needs. The assembled electronic elements are located inside a light-absorbent CASE topped with a ring-shaped holder (DISH HOLDER) where the sample dish is placed at a distance of 1 cm from the LED board and irradiated from below. During irradiation experiments, the culture dish was covered with a LID to avoid light scattering.

Bacterial suspensions were irradiated by means of a customized LED-based illuminating prototype. Selected LEDs (Nichia Corporation Ltd., Tokushima, Japan) were mounted on a board coupled with a programmable microcontroller (LPC81xM, NXP Semiconductors); two lithium batteries (CR2, 3 V) provide suitable power according to experimental needs. The assembled electronic elements are located inside a light-absorbent case (4 cm × 4 cm; height: 2.5 cm), topped with a ring-shaped holder where the sample dish is placed at a distance of 1 cm from the LED board and irradiated from below (Figure 1). Before the experiment the device was programmed (*via* a dedicated software) to set the desired control current, the number and position of the active LEDs and the irradiation time. Four different boards were used, each one mounting identical LEDs, whose number and position were designed to obtain comparable light intensities and a homogeneous irradiation field on the sample. The selected LEDs were: NVSU233A-U405 (Violet, 405 nm, FWHM ≈ 12 nm, 8 LEDs per board), NSSC146AT (Blue, 460 nm, FWHM ≈ 18 nm, 40 LEDs per board), NCSE119BT-V1 (Bluish-Green, 500 nm, FWHM ≈ 30 nm, 8 LEDs per board), NCSR219BT-V1-E (Red, 630 nm, FWHM ≈ 16 nm, 8 LEDs per board). These LEDs were selected to match the Soret band (405 nm), the shortest (500 nm), and the longest wavelength (630 nm) Q bands of porphyrin absorption spectrum and a spectral range (460 nm) where the porphyrin absorption spectrum has a minimum.

Control measurements were performed to assure the stability of the control current, and consequently of the irradiance, in the time range of the designed experiments. Furthermore, preliminary tests allowed the determination of the maximum control current and the appropriate time extent of the irradiation in order to keep the sample temperature in the physiological range. Control current values were tested in steps of 25 mA up to 100 mA and the corresponding irradiance values, used throughout the experiments, were measured using a light spectroradiometer (SAMA Tools by S.A.M.A Italia Srl, Lucca, Italy) (Battisti et al., 2018).

### Photoinactivation Assays

For the photoinactivation assays, an aliquot (2 ml) of a bacterial suspension (2 × 10^6^ CFU/ml) was transferred to a Petri dish (35 mm) and irradiated in aerobic atmosphere at room temperature, according to the absorption spectrum of *H. pylori* endogenous porphyrins. At least three independent replicates were performed for each experiment. Following light irradiation assays, the illumination efficacy was assessed in comparison with the dark control by plating serial dilutions of each sample on Brucella agar plates with 10% FBS as described previously (Jett et al., 1997). Plates were incubated at 37°C in microaerophilic atmosphere for 4 days. After incubation, surviving bacterial cells (CFU/ml) were counted and, when indicated, survival fractions were determined relatively to untreated bacterial suspensions and expressed as Log CFU/ml.

### Scanning Electron Microscopy

In order to examine the effects of irradiation at 405 nm on bacterial surface morphology, non-irradiated and irradiated samples were observed by scanning electron microscopy. A bacterial suspension of *H. pylori* ATCC 43504 (1 × 10^7^ cells/ml) was exposed to sublethal light dose at 405 nm (9.3 J/cm^2^), as described above. Then, non-irradiated and irradiated samples were centrifuged and the pellet was fixed with 2% glutaraldehyde and 1% paraformaldehyde in 0.1 M cacodylate buffer for 1 h at room temperature and then overnight at 4°C. Samples were washed three times for 5 min in 0.1 M cacodylate buffer and postfixed in 1% OsO_4_ and 1% K_3_Fe(CN)_6_. After washing with distilled water, samples were dehydrated through increasing ethanol concentrations (30%, 50%, 70%, 90%, and 100%) in sequential steps of 10 min incubation. Finally, an aliquot of each sample was transferred on a silicon wafer, dried at the critical point and sputter coated with gold (8 nm) before imaging.

### Fluorescence Characterization of *H. pylori* Extracts

Bacterial extracts from *H. pylori* cultures (100 ml) were prepared as previously described (Battisti et al., 2017a, b). *H. pylori* cells were harvested by centrifugation (7000 × *g* at 4°C for 10 min), washed in 20 ml pre-chilled buffer (0.05 M Tris pH 8.2–2 mM EDTA) and suspended in 10 ml of the same buffer. An aliquot (1.5 ml) of a mixture of ethyl acetate and acetic acid (3:1, v/v) was added and bacterial cells were lysed by sonication in ice. Then, the organic phase was extracted with 100 μl of HCl 3 M. After vigorous vortexing, this solution was centrifuged (7000 × *g* for 5 min) and then the bottom layer was collected for spectroscopic analysis and diluted in HCl 3M/MeOH 1:3. Fluorescence measurements were carried out with a Cary Eclipse fluorometer (Varian, Palo Alto, CA, United States) using 5 nm excitation band-pass, 5 nm emission band-pass, 0.5 s integration time.

### High Performance Liquid Chromatography and Mass Spectrometry

High Performance Liquid Chromatography-Mass Spectrometry analyses were performed on a Shimadzu Nexera UHPLC chromatograph interfaced with an Ab Sciex 3200 QTRAP mass spectrometer (AB SCIEX, Toronto, ON, Canada). HPLC analyses were performed using a Phenomenex Kinetex PFP column (3 × 150 mm) using water/formic acid 100/0.1 v/v (A) and acetonitrile/formic acid 100/0.1 v/v (B) as mobile phases at 0.8 ml/min flow. Runs were performed under the following conditions: 2 min at 25% B, then a linear gradient to 95% B in 26 min, followed by a 4-min purge step at 95% B and by a 8-min re-equilibration step to the starting conditions. MS analyses were performed under the following conditions: ion spray voltage: 5000 V, source temperature 350°C, declustering potential 50 V, ion source gas 20 l/min, curtain gas: 25 l/min. m/z ratios between 200 and 1300 were monitored.

### Statistical Analysis

The results of photoinactivation were expressed as mean ± standard error of the means (SEM). Differences between illuminated and unilluminated samples were evaluated with one-way ANOVA test, followed by Tukey-Kramer *post hoc* test using GraphPad Instat software (version 6.05 for Windows, La Jolla, CA, United States). The level of significance was set at a *P* value of <0.05.

## Results

### *H. pylori* Photoinactivation at 405 nm

A first set of measurements was performed irradiating the two *H. pylori* strains (ATCC 43504 and the virulent ATCC 700824) at 405 nm, the most effective wavelength in bacterial photokilling (Hamblin et al., 2005). Samples were exposed to 8.64 mW/cm^2^ irradiance (corresponding to the control current value of 25 mA) for times ranging between 5 and 20 min thus administering the following light doses: 2.59, 5.18, 7.78, and 10.4 J/cm^2^.

**FIGURE 2:**
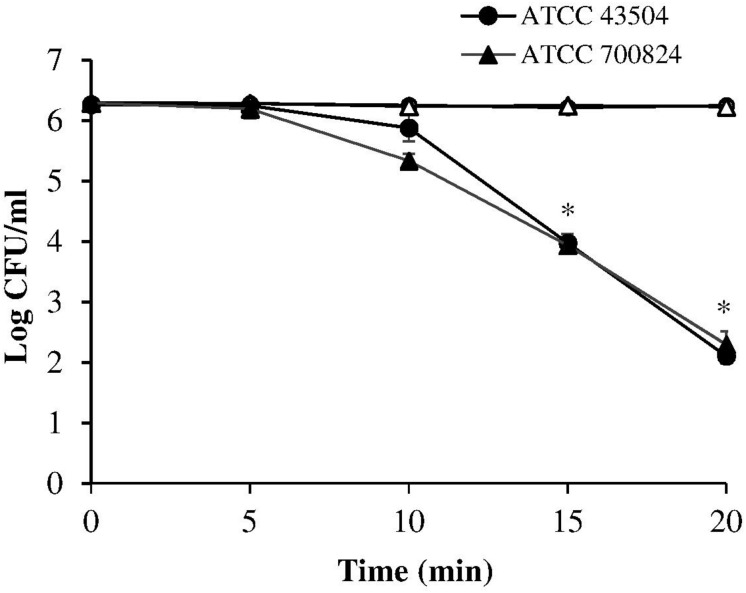
Photoinactivation at 405 nm. *H. pylori* ATCC 43504 and ATCC 700824 were illuminated at 405 nm (8.64 mW/cm^2^ irradiance) for different exposure time points in aerobic atmosphere at room temperature (closed circle and closed triangle for ATCC 43504 and ATCC 700824, respectively). Data are expressed as means of three independent experiments ±SEM. ^∗^ *P* < 0.001, as compared to the dark control (open symbols).

As shown in Figure 2, the viability of both the tested *H. pylori* strains was similarly affected by the 405 nm irradiation. A significant photoinactivation effect was quickly achieved after 15 min illumination (7.78 J/cm^2^), resulting in a 2.3 Log reduction in cell viability of both *H. pylori* strains, as compared to the dark control. The photoinactivation effect further increased (*P* < 0.001) after 20 min of light exposure (10.4 J/cm^2^), causing about four Log reduction of bacterial count.

A reciprocity test of the photoinactivation effect was then performed on the ATCC 700824 strain administering various radiation doses at 405 nm, varying both irradiance and exposure time, as shown in Table 1.

**TABLE 1:** Irradiance and exposure time values for the reciprocity test.

Dose (J/cm^2^)	Time (sec)		
	
	40.7 mW/cm^2^ (100 mA)	31 mW/cm^2^ (75 mA)	21.2 mW/cm^2^ (50 mA)
2.6	64	84	123
6.4	157	206	302
9.3	229	300	439
12.2	300	394	575

**FIGURE 3:**
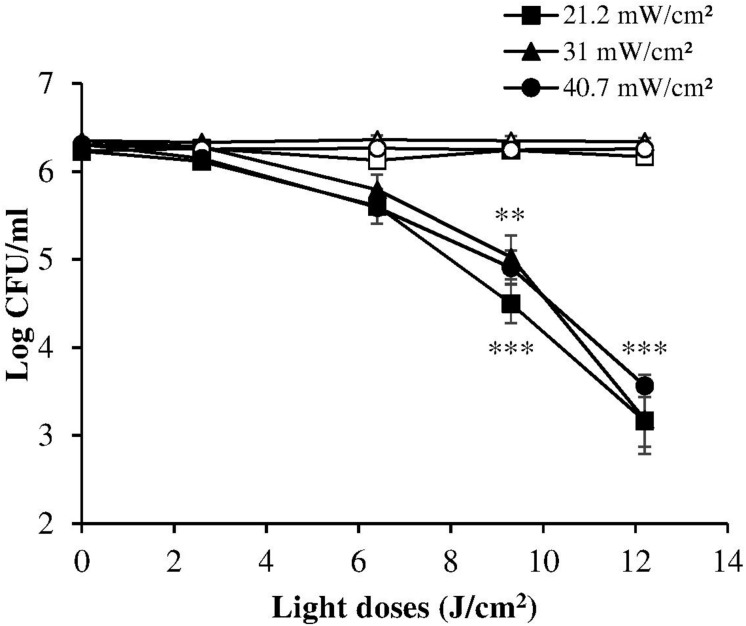
Comparison between dose-effect curves of *H. pylori* photoinactivation at 405 nm at three different irradiances. *H. pylori* ATCC 700824 (about 2 × 10^6^ CFU/ml) was exposed to different light doses (405 nm) delivered at constant irradiances (21.2 mW/cm^2^, closed square; 31 mW/cm^2^, closed triangle; 40.7 W/cm^2^, closed circle), under aerobic atmosphere, changing the exposure time (see Table 1), thus providing the same light dose. Data are expressed as means of at least three independent experiments ±SEM. ^∗∗^ *P* < 0.01; ^∗∗∗^ *P* < 0.001, as compared to the dark control (open symbols).

The dose-response curves at the three irradiance values (reported in Figure 3, together with the results of the corresponding control measurements) show a very similar trend, indicating that in these experimental conditions the photoinactivation effect is dose-dependent regardless of the administration method (i.e., varying irradiance or irradiation time).

### Dose-Effect Curves at Different Wavelengths

**FIGURE 4:**
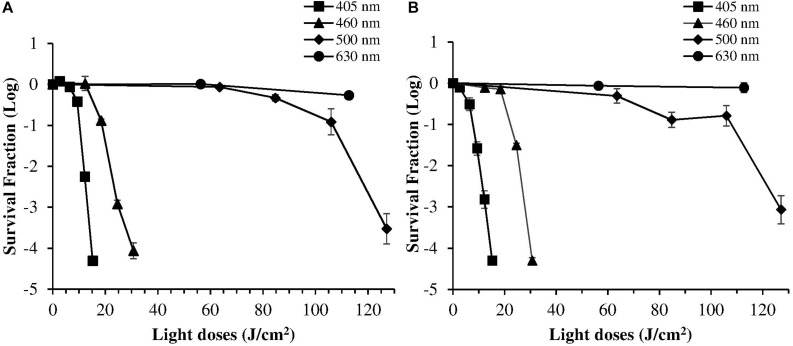
Comparison of photoinactivation curves of *H. pylori* ATCC 43504 **(A)** and ATCC 700824 **(B)** strains following exposure to light at different wavelengths: 405 nm (square), 460 nm (triangle), 500 nm (diamond), 630 nm (circle). *H. pylori* cells (about 2 × 10^6^ CFU/ml) were illuminated at room temperature under aerobic atmosphere. Data are expressed as means of survival fraction of at least three independent experiments ±SEM.

**FIGURE 5:**
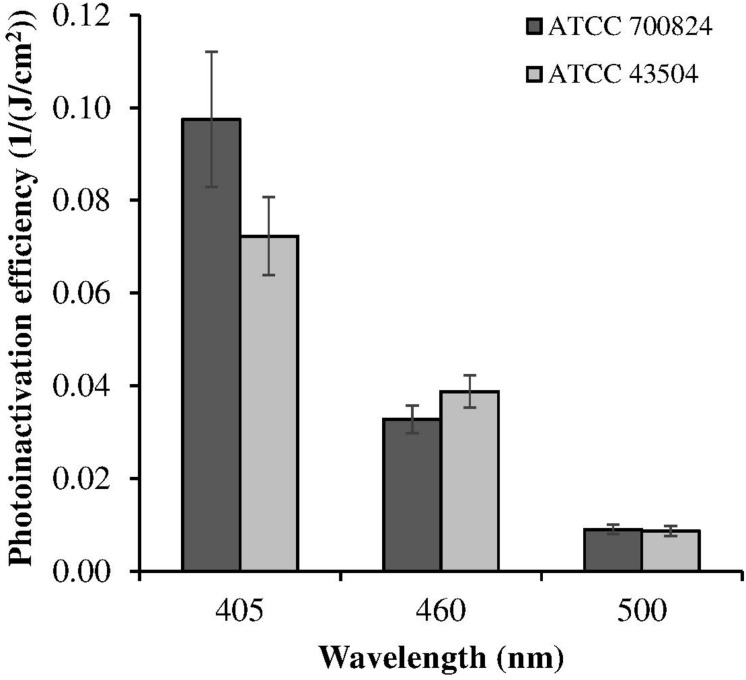
Relative photoinactivation efficiency at different wavelengths for *H. pylori* ATCC 43504 and ATCC 700824 strains. Photoinactivation efficiency is plotted as the reciprocal of the light dose required to induce a three Log reduction in bacterial viability.

Despite violet light may cause an efficient *H. pylori* photokilling *in vitro*, its effects may be limited to the most superficial layers of the gastric mucosa because of its low penetration through tissues, thus probably hampering the eradication of the infection *in vivo*. Porphyrins also have absorbance bands in the green and red spectral regions, and light of those wavelengths penetrates deeper into the layers of the gastric mucosa. Thus, in order to choose the most effective combination of wavelengths to implement inside the ingestible LED-equipped pill, dose-effect curves obtained by exposure of *H. pylori* at different wavelengths corresponding to the peaks of the Soret (405 nm) as well as Q (500 and 630 nm) porphyrin absorption bands were compared. As negative control, irradiation was also performed at 460 nm where porphyrin absorbance has a minimum (Figures 4A,B). Figure 5 shows for the two bacterial strains the relative photokilling efficiency (action spectrum) at 405 nm, 460 nm and 500 nm, plotted as the reciprocal of the light dose required to induce a 3 Log reduction in bacterial viability, which is conventionally regarded as bactericidal (Nakonieczna, 2017). These values were estimated by linear interpolation of the appropriate data points of the dose-effect curves. The action spectrum indicates that for the virulent strain ATCC 700824 the photoinactivation effect at 405 nm was about 3 and 10 times higher than the one observed at 460 nm and at 500 nm, respectively, whereas it is about 2 and 7 times higher for the laboratory-adapted strain ATCC 43504.

The irradiation at 630 nm did not induce any photoinactivation effect, even using the maximum light dose (maximum exposure time of 1 h; LED at 630 nm; 31.3 mW/cm^2^ irradiance) allowed without reaching non-physiological temperatures.

### Effect of Irradiation on *H. pylori* Cell Morphology

**FIGURE 6:**
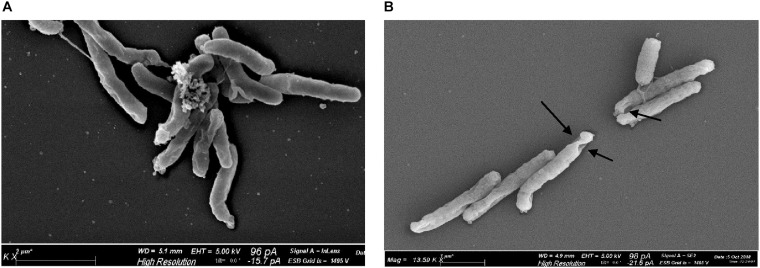
Effect of illumination at 405 nm on bacterial morphology. Representative scanning electron microscope images of *H. pylori* ATCC 43504 (5 × 10^7^ cells/ml): **(A)** non-irradiated and **(B)** irradiated cells at 405 nm (9.3 J/cm^2^). About 100 cells/sample were analyzed. Arrows indicate the presence of holes on the surface of irradiated bacteria.

Scanning electron microscopy revealed morphological alterations on the bacterial surface of *H. pylori* cells, exposed to the sublethal light dose of 9.3 J/cm^2^ at 405 nm, affecting the cell viability without inducing a massive reduction of bacterial count. Non-irradiated bacteria, used as control, showed a curved or spiral shape and their bacterial surface appeared intact, with one or a few flagella at one end (Figure 6A). Conversely, irradiated *H. pylori* cells were damaged showing holes on the bacterial surface (Figure 6B), evidence of cellular suffering and prelude to bacterial death. No coccoid form was observed in both non-irradiated and irradiated cells.

### Spectroscopic Characterization of Endogenous Photosensitizers

**FIGURE 7:**
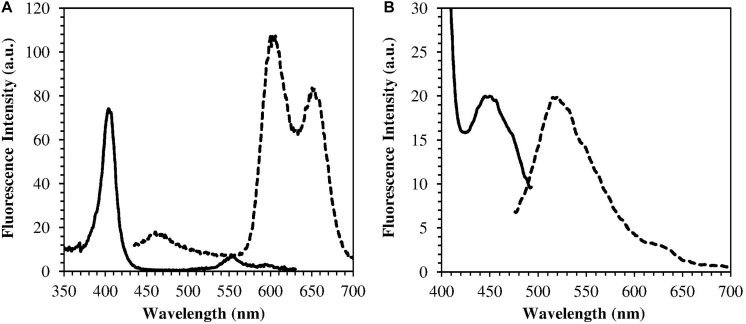
Emission (dotted curves) and excitation (solid curves) fluorescence spectra of *H. pylori* extracts. **(A)** l_exc_ = 405 nm, l_em_ = 660 nm (porphyrin fluorescence); **(B)** l_exc_ = 460 nm, l_em_ = 520 nm (flavin fluorescence).

**FIGURE 8:**
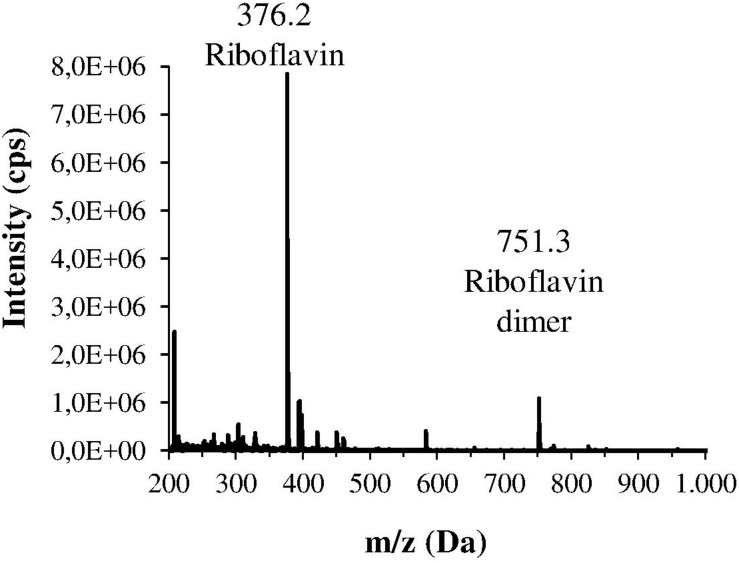
Mass spectrum of one the main fractions of the bacterial extract, isolated by HPLC. This spectrum corresponds to the fraction with the lowest retention time, and shows an intense m/z peak at 376.2 Da and a lower peak at 751.3 Da that could suggest the presence of riboflavin and its dimer.

The *H. pylori* photoinactivation observed at 460 nm (Figure 5) is too high to be exclusively attributed to the photosensitizing action of endogenous porphyrins. In fact, on the basis of porphyrin spectroscopic features, the difference in light absorption at 405 and 460 nm should correspond to a similar difference in bacterial photosensitivity: a tenfold reduction of the effect was expected, but only a two-threefold reduction was obtained by shifting the irradiation from 405 nm to 460 nm. In order to identify a second putative endogenous photosensitizer, fluorescence emission and excitation spectra were performed on bacteria extracts. In Figure 7A the fluorescence emission (l_exc_ = 405 nm) and excitation (l_em_ = 660 nm) spectra showed the typical spectroscopic features of porphyrins, as expected (Battisti et al., 2017a). Exciting *H. pylori* extracts at 460 nm, a broad fluorescence emission band peaked around 525 nm was observed while no porphyrin emission bands at 600 nm and 650 nm were detected (Figure 7B). As in the case of other photosensitive bacteria the broad fluorescence peak around 525 nm can be assigned to flavin compounds, namely, riboflavin, FAD, and FMN, which exhibit very similar absorption and emission characteristics (Plavskii et al., 2018). Along with the flavin-like fluorescence emission, an intense peak in the mass spectrum of the bacterial extracts, corresponding to an m/z ratio of 376, suggests the presence of riboflavin as a significant component of the mixture (Figure 8).

## Discussion

The present study shows that visible light irradiation by using an innovative LED-based device can efficiently induce *in vitro* photokilling in *H. pylori* without the administration of exogenous photosensitizers. In fact, a significant reduction of cell viability was observed after illumination with LEDs at 405 nm on both tested *H. pylori* strains, including the virulent one, ATCC 700824. In addition, the induced photokilling effect is mainly dependent on the delivered light dose, at least within the tested values range. Indeed, combinations of longer exposure times with lower irradiances, or shorter exposure times with higher irradiances, provided a similar photokilling effect. Thus, according to the reciprocity law of Bunsen-Roscoe, the observed photoinactivation is directly proportional to the total energy dose, regardless of the administering regime (Schindl et al., 2001). Although few data are currently available on this issue, reciprocity is often found to hold true in bacteria, unlike in eukaryotic organisms endowed with more complex mechanisms of oxidative damage repair (Farr and Kogoma, 1991; Schindl et al., 2001; Morita et al., 2010; Taraszkiewicz et al., 2015; Ogonowska et al., 2019). Considering the potential clinical application of aPDT, this aspect is crucial in defining the most effective protocol of irradiation, and thus it should be taken into account for *in vitro* aPDT studies.

As previously referred by Hamblin et al. (2005), also in our irradiation experiments 405 nm was the most effective wavelength in inducing photokilling in both *H. pylori* strains. Much higher doses of 460 nm and 500 nm wavelengths were necessary to induce a bactericidal effect, whereas the 630 nm irradiation was ineffective. Therefore, in accordance with other studies, the efficacy of bacterial photoinactivation at different wavelengths mainly correlates with the light absorption spectrum of endogenous porphyrins, decreasing with the increase in irradiation wavelength (Pummer et al., 2017). However, the observed photoinactivation of *H. pylori* by light of 450–470 nm is much higher than expected on the basis of porphyrin absorption characteristics, since they only exhibit a strong absorption in the spectral region of 380–420 nm and a weak absorption in the 500–650 nm range. Previous studies on other microorganisms demonstrated that significant bacterial photoinactivation around 460 nm is mediated by flavin-like molecules (Hessling et al., 2017; Hoenes et al., 2018; Tomb et al., 2018). Our spectroscopical and mass analysis studies of bacterial extracts indicate for the first time flavin compounds, along with porphyrins, as endogenous photosensitizers responsible for *H. pylori* photoinactivaction.

As far as the cellular targets are concerned, our scanning electron images show morphological alterations of the bacterial cell wall after administration of a sub-lethal light dose. This could be the main damage of the photodynamic action, leading to bacterial death through leakage of cellular contents or inactivation of membrane transport systems and enzymes (Caminos et al., 2008; Ma et al., 2018).

As reported by safety studies on the side effects of aPDT on host cells and tissue, our irradiation conditions are sufficient to inactivate *H. pylori*, but are ineffective to cause lethal oxidative bursts through excitation of endogenous pigments of mammalian cells (Wang et al., 2017).

In conclusion, the overall findings suggest that aPDT could be an alternative safe effective treatment, or an adjuvant therapy to conventional antibiotics for *H. pylori* eradication. Cost-effective and energy-efficient LEDs emitting in the violet (405 nm) and blue (460 nm) region could efficiently photoinactivate *H. pylori* and can be integrated in final ingestible pills used for gastric irradiation. The endogenous photosensitizers responsible for bacterial photokilling are not only porphyrins, PPIX and CPI and III, but also flavin-type molecules such as riboflavin.

Further studies will be aimed at evaluating the efficacy of irradiation on biofilms produced by clinical *H. pylori* strains since bacteria embedded therein may exhibit different characteristics as compared to their planktonic counterparts in terms of resistance toward antimicrobials (Costerton et al., 1995; Hathroubi et al., 2018).

## Data Availability Statement

The raw data supporting the conclusion of this article will be made available by the authors, without undue reservation, to any qualified researcher.

## Author Contributions

PM, FG, and AS conceived and designed the experiments. PM, AB, GT, and AS performed the experiments. PM, AB, FG, GC, and AS analyzed the data. PM drafted the manuscript, with the contribution of AB, GC, FG, and AS. All authors reviewed and revised the first and final drafts of this manuscript.

## Conflict of Interest

The authors declare that the research was conducted in the absence of any commercial or financial relationships that could be construed as a potential conflict of interest.
